# Can Artificial Intelligence “Hold” a Dermoscope?—The Evaluation of an Artificial Intelligence Chatbot to Translate the Dermoscopic Language

**DOI:** 10.3390/diagnostics14111165

**Published:** 2024-05-31

**Authors:** Emmanouil Karampinis, Olga Toli, Konstantina-Eirini Georgopoulou, Elli Kampra, Christina Spyridonidou, Angeliki-Victoria Roussaki Schulze, Efterpi Zafiriou

**Affiliations:** 1 Department of Dermatology, Faculty of Medicine, School of Health Sciences, University General Hospital of Larissa, University of Thessaly, 41110 Larissa, Greece; kampraelli@gmail.com (E.K.); roussaki@otenet.gr (A.-V.R.S.); 2 Department of Dermatology, Oncoderm Center One Day Clinic, 45332 Ioannina, Greece; olgatolimail@gmail.com; 3 Department of Dermatology, General Hospital of West Attica “Agia Varvara”, 12351 Athens, Greece; koneirgeo@gmail.com; 4 Department of Dermatology, Athens Naval Hospital, 11521 Athens, Greece

**Keywords:** artificial intelligence, chatbots, dermoscopy, differential diagnosis

## Abstract

This survey represents the first endeavor to assess the clarity of the dermoscopic language by a chatbot, unveiling insights into the interplay between dermatologists and AI systems within the complexity of the dermoscopic language. Given the complex, descriptive, and metaphorical aspects of the dermoscopic language, subjective interpretations often emerge. The survey evaluated the completeness and diagnostic efficacy of chatbot-generated reports, focusing on their role in facilitating accurate diagnoses and educational opportunities for novice dermatologists. A total of 30 participants were presented with hypothetical dermoscopic descriptions of skin lesions, including dermoscopic descriptions of skin cancers such as BCC, SCC, and melanoma, skin cancer mimickers such as actinic and seborrheic keratosis, dermatofibroma, and atypical nevus, and inflammatory dermatosis such as psoriasis and alopecia areata. Each description was accompanied by specific clinical information, and the participants were tasked with assessing the differential diagnosis list generated by the AI chatbot in its initial response. In each scenario, the chatbot generated an extensive list of potential differential diagnoses, exhibiting lower performance in cases of SCC and inflammatory dermatoses, albeit without statistical significance, suggesting that the participants were equally satisfied with the responses provided. Scores decreased notably when practical descriptions of dermoscopic signs were provided. Answers to BCC scenario scores in the diagnosis category (2.9 ± 0.4) were higher than those with SCC (2.6 ± 0.66, *p* = 0.005) and inflammatory dermatoses (2.6 ± 0.67, *p* = 0). Similarly, in the teaching tool usefulness category, BCC-based chatbot differential diagnosis received higher scores (2.9 ± 0.4) compared to SCC (2.6 ± 0.67, *p* = 0.001) and inflammatory dermatoses (2.4 ± 0.81, *p* = 0). The abovementioned results underscore dermatologists’ familiarity with BCC dermoscopic images while highlighting the challenges associated with interpreting rigorous dermoscopic images. Moreover, by incorporating patient characteristics such as age, phototype, or immune state, the differential diagnosis list in each case was customized to include lesion types appropriate for each category, illustrating the AI’s flexibility in evaluating diagnoses and highlighting its value as a resource for dermatologists.

## 1. Introduction

Artificial intelligence (AI) is increasingly becoming popular in the section of dermatology and has become a helping hand to dermatologists for skin cancer diagnosis as well as difficult cases. Currently, studies report high accuracy performance, even exceeding dermatologists for the diagnosis of skin lesions [1]. The use of artificial intelligence in dermatology is mainly image-based and is constructed by the deep learning (DL) methodology, which makes connections between inputs such as images and outputs such as diagnoses like BCC or melanoma [2]. As a result, a complex mapping is created with links that bond image patterns and prediction of diagnosis. Deep learning is usually based on self-learning and training through image databases that contain macroscopic or dermatoscopic images of skin cancer subtypes [1,3]. The convolutional neural networks (CNNs), the leading DL algorithm for image analysis, have reported a high performance rate in melanoma diagnosis [4]. Additionally, due to the unpredictable nature of inflammatory dermatoses [5], AI technology has been able to predict the flares of skin diseases such as psoriasis and atopic dermatosis [6].

Apart from the image-based and flare-up-predicting AI, which is the hallmark of this type of technology in dermatology, chatbots are emerging as a new addition to the dermatological domain. They offer a convenient and practical tool that is accessible not only to clinicians at all levels of healthcare but also to patients. AI chatbots represent a novel type of artificial intelligence that employs natural language comprehension techniques in conversations with users when they pose a question. The methodology used by chatbots is also based on neural networks that are trained on extensive text datasets [7]. Dermatology is a domain that includes complicated terminology, ranging from complicated dermatosis presentations, including primary and secondary cutaneous lesions, to the dermoscopic language and dermatopathological reports. Therefore, how efficiently can a text-based AI program such as a chatbot deal with questions regarding this “cutaneous” aspect of medicine?

OpenAI’s ChatGPT is a program built by the chatbot model technology. The advanced search of the combination of (ChatGPT) AND (medicine) results in more than 1500 studies over a period of only 2 years. The chatbot operates on GPT-3.5 or GPT-4 (if available through subscription). GPT-4, the most recent model released by OpenAI, boasts advancements over its predecessor (ChatGPT-3.5). It is reported to excel in tackling more complex problems with greater accuracy, attributed to enhanced reasoning capabilities and a significantly expanded repository of learned information. However, GPT-3.5 is more popular due to the lack of a need for a subscription [8,9].

Chatbots including GPT-3.5 and GPT-4 have been frequently used in dermatology, usually for teaching purposes and patient confrontation. Most of the studies aim to present complicated medical issues in a simplified language for the patients to understand or even lead to self-diagnosis. ChatGPT can be helpful in aiding healthcare providers in formulating differential diagnoses and therefore aid practitioners, and, more frequently, new-certified clinicians, to formulate their approach to the patients. This approach is also expanded in dermatology [9]. The differential diagnoses generated are also in question regarding their completeness and contribution to diagnosis. ChatGPT can become virtual tutors and be tailored to each student’s unique learning preferences and aptitudes. Additionally, ChatGPT can generate dermatological scenarios and support students in their academic endeavors by addressing their queries and crafting summaries about specific dermatological themes [10]. 

Chatbots have been utilized and investigated across various domains of dermatology, as outlined in Box 1, encompassing tasks such as skin cancer detection, the monitoring of inflammatory dermatoses, and applications in aesthetic medicine. Box 1 includes studies published until 28 March 2024 on Pubmed regarding the use of chatbots in dermatology. Specifically, patients often inquire about symptoms or the appearance of lesions they are experiencing prior to consulting a physician, seeking to determine if they could signify malignancies or systemic illnesses [9]. If the responses from the chatbot suggest the possibility of cancer or serious conditions, patients typically seek medical attention [10]. Additionally, patients frequently seek further information about their condition, including its etiology, adherence to treatment, whether it is contagious, and how it may impact their daily lives. Also, disease-specific counseling is considered as virtual healthcare assistants available at any time, such as for hidradenitis suppurativa [11]).

Box 1 **Box presenting the use of chatbots in different aspects of Dermatology practice.**

Use of chatbots in dermatology

- **Skin cancers**

ChatGPT generation of medical information in response to 25 clinical questions about NMSC (non-melanoma skin cancer): use of language learning models and zero-shot chain of thought (“Let’s think step by step!” strategy) [12].

ChatGPT4 reproduced dermatohistopathological reports in a patient-friendly language effectively [13].

- **Inflammatory Dermatosis**

**Psoriasis**

Investigation of the abilities of ChatGPT to compare the different systemic therapeutic interventions for moderate-to-severe psoriasis [14].

**Atopic Dermatitis**

By comparing ChatGPT answers regarding atopic dermatitis and acne, precision and inclusiveness were diminished for inquiries regarding acne treatment compared to atopic dermatitis (AD), occasionally omitting details on treatment efficacy, lacking clarity on treatment outcomes, and neglecting to offer tailored recommendations suitable for the patient’s age and individual circumstances [15].

**Rosacea**

ChatGPT 3.5 demonstrates remarkable reliability and practical relevance when addressing common inquiries from patients regarding rosacea, as evidenced by the analysis of 20 questions–answers [16].

**Hidradenitis**

A medical hidradenitis chatbot can provide high-quality information within seconds [11].

**Allergy**

The effective utilization of a chatbot to streamline and automate the process of obtaining medical history in cases of Hymenoptera venom allergy [17].

**Aesthetic Medicine**

A text-based patient information chatbot named ‘Beautybot’ covering themes such as wrinkles and pigmentation disorders. Written and image-based content was provided in a multiple-choice-driven algorithmic workflow [18].

Chatbots have been examined with radiological [19] and histopathological languages, with interesting translation results. Both studies reported the beneficial effect of chatbots on those patients; the clinicians tried to understand the two different medical languages, and the respective clinicians confirmed their usefulness. Regarding the reports on dermatopathology, the majority of physicians either agreed or strongly agreed that ChatGPT-4 interpretations of the reports were thorough, precise, comprehensible, and unlikely to result in harm, while there was no variance in physicians’ assessments of translations between inflammatory and neoplastic diagnoses [13]. On the other hand, dermoscopy is a rather complicated language, and to our knowledge, no study has examined the ability of a chatbot to interpret dermoscopic descriptions. 

Dermoscopy is a widely utilized noninvasive diagnostic method that enhances the accuracy of diagnosing pigmented and cancer-suspicious lesions compared to naked eye examination. The dermoscopy-related vocabulary is specialized. Many terms within this lexicon are metaphorical, such as the “starburst” pattern, or signs such as the “delta glider sign”. The descriptive terms are used to describe pigment distribution (for example, dots and leaf-like structures), vessel morphology (arborizing and dotted vessels), and keratin disturbances. Although descriptive terms and metaphors can assist in remembering and establishing connections between particular dermoscopic features and diagnoses, ambiguity stemming from identical descriptive terms for the same structure can pose a potential obstacle to both learning and research within the field. As a result, it is reasonable to wonder how a chatbot can deal with the understanding and creation of such specialized vocabulary [20].

Therefore, it is reasonable to assess the ability of a chatbot to enhance clinicians’ understanding and proficiency in dermoscopic examination through interactive, personalized, and informative interactions.

## 2. Materials and Methods

This study employed a survey-based methodology to assess the performance and utility of an artificial intelligence (AI) chatbot in translating dermoscopic descriptions of skin lesions, supplemented with specific clinical information such as age, skin tone, and immunocompetency. The AI chatbot utilized for this research was ChatGPT 3.5, recognized as one of the most commonly used chatbots in everyday settings. The inclusion criteria comprised individuals certified in the dermoscopic interpretation and diagnosis of skin lesions (dermatologists or doctors with a certification in dermoscopy such as an MSc).

A structured survey was designed to evaluate the differential diagnosis provided by the AI chatbot. The survey assessed the completeness and diagnostic utility of the chatbot-generated reports, focusing on their contribution to accurate diagnosis and educational opportunities for novice dermatologists. Specific attention was given to potential inaccuracies or misleading conclusions in the differential diagnosis. Participants were presented with a series of hypothetical dermoscopic descriptions of skin lesions, each accompanied by specific clinical information. They were subsequently requested to assess the differential diagnosis list generated by the AI chatbot in its initial response, without including the accompanying text or information, such as the descriptions of the listed lesions or recommendations for visiting a dermatologist. Following each presentation, the participants were prompted to provide feedback on the completeness, accuracy, and utility of the chatbot-generated differential diagnosis. The correct answers were not revealed to the participants at the time of evaluation.

Participation in this study was voluntary. The survey instrument ensured anonymity of responses, and data confidentiality was strictly maintained throughout this study.

Each question was marked using a scale of 1 (disagree) to 3 (agree), and depending on the answers of the clinician on each chatbot’s differential diagnosis, each case would obtain an average score. The average scores on a questionnaire would be collected and compared in each case. The survey comprised three instances of dermoscopic descriptions, covering basal cell carcinoma (BCC), squamous cell carcinoma (SCC), benign lesions, pigmented lesions, and inflammatory dermatoses in each case. It also included descriptions of signs, along with three scenarios detailing age, lesion location, phenotype, and immune state specifications. The total score comprised the average value and a standard deviation. The average values were reported to one decimal place, while standard deviation values were reported to two decimal places. The comparison of scores between more than two groups was performed using a one-way analysis of variance test with post-hoc multiple two-group comparisons for normal data, and a Kruskal–Wallis test was used for non-normal data. To compare categorical data, the χ^2^-test was performed. A Shapiro–Wilk test was used to test for normality.

## 3. Results

A total of 30 clinicians with dermoscopic knowledge, who were eligible according to our inclusion criteria, completed the questionnaire by judging the answers the chatbot gave. The majority of participants (83.3%) were female professionals working across various regions in Greece, including both rural and urban areas, as well as in hospitals and health centers. Among them, 40% were clinicians with an MSc in dermoscopy, 20% were dermatology residents, and 40% were experienced dermatologists with over twenty years of clinical practice, collectively encompassing a broad spectrum of dermoscopic expertise.

**Figure 1:**
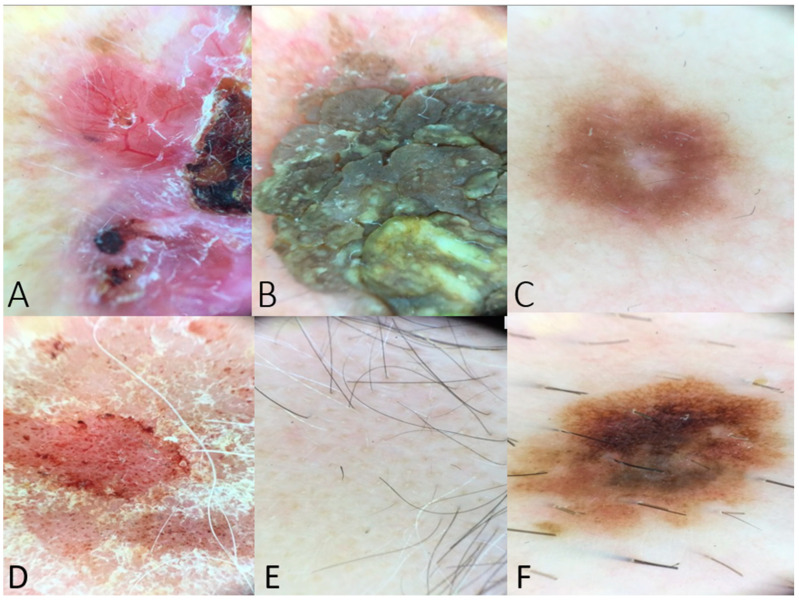
(**A**) Dermoscopy of a BCC lesion including arborizing vessels, ulceration, shiny white-red structureless areas, and short white streaks (example used in Table 1). (**B**) Dermoscopy image of seborrheic keratosis with fissures and ridges giving cerebriform appearance (example used in questions regarding dermoscopic features of benign lesions). (**C**) Peripheral delicate pigment network and central white patch in case of dermatofibroma (used as a question to the chatbot). (**D**) Dermoscopy image of psoriasis lesion with distributed dotted vessels in a reddish pink background and white scale. (**E**) Yellow and black dots and at least one broken hair in a case of alopecia areata (example posed to the chatbot in the category of inflammatory dermatoses). (**F**) Dermoscopy image indicating an atypical nevus with an atypical pigment network, asymmetry in structure or color, brown dots, and irregular dots and globules. This image was posed as a scenario to the chatbot, and the differential diagnosis was assessed.

The hypothetical dermoscopic scenarios included descriptions of skin lesions with classic findings (Figure 1). 

The initial set of queries to the chatbot comprised dermoscopic images associated with BCC. Within two of the descriptions provided, the characteristic vascular pattern of BCC, known as arborizing vessels, and ulceration, a common feature in BCCs, were observed [21]. BCC was listed in all of the chatbot’s differential diagnoses. Additionally, the list included other skin cancers like SCC and melanoma, as well as a more general category termed “other dermatological conditions”. However, the chatbot also included diagnoses that were inconsistent with the provided description, such as atypical nevus, as no pigmented network was reported in the description. Despite these variations, the lists in all the examples received high scores from the assessing dermatologists (Table 1).

The next triad was about SCC-based dermoscopic images with the description involving classic dermoscopic features encountered in SCC such as the pink background, white circles, and glomerular vessels. Also, different types of SCC were described as the second description is in situ SCC (Bowen’s disease), and when the homogenous pigmentation is included, the lesion is more likely to be a pigmented Bowen disease [22]. The chatbot’s differential diagnosis list includes Bowen’s disease, along with pigmented BCC, actinic keratosis, sebaceous hyperplasia, and other potential conditions. Amongst the descriptions, no statistically significant differences were noticed performing Kruskal–Wallis statistics in the “complete” (*p* = 0.128 > 0.05) category. In the category of “helpful to diagnosis,” a statistically significant difference was observed between descriptions 1 and 3 (post-hoc Dunn’s test: *p* = 0.045 < 0.05), potentially because the provided description is broader and the differential diagnosis included is more extensive. This pairing assists clinicians in making diagnoses more efficiently. Moreover, the lack of correct answers in the list for the first SCC description results in reduced scores for dermatologists. It is worth mentioning that both the first and third descriptions stem from the good differentiation of SCC, indicating the same type of SCC. A similar pattern of scores was followed in the “teaching tool” category (*p* = 0.01 < 0.05), with statistical significance between the 1st and 2nd descriptions (post-hoc Dunn’s test, *p* = 0.013) and 1st and 3rd descriptions (post-hoc Dunn’s test, *p* = 0.006) due to the bad performance of the chatbot in the 1st description, not including the correct answer (Table 2).

Benign lesion-based images triad were based on dermoscopic descriptions of actinic and seborrheic keratosis and dermatofibroma. The dermoscopic characteristics of those are classic and frequently used in dermoscopic algorithms to exclude skin malignancies [23,24,25]. The correct answer was included in all the answers in this triad, while the differential diagnosis was wide, including inflammatory dermatoses, apart from skin cancer and benign neoplasms (Table 3). Also, the 2nd description, which was represented by seborrheic keratosis, did not include signs or vessels but a shape characterization (cerebriform appearance). In this case as well, the chatbot found the correct answer and formed a satisfactory differential diagnosis according to the dermatologists-participants in the survey. Statistical significance in the satisfaction of the participants was observed with actinic keratosis achieving the lower score (Kruskal–Wallis test between the groups; groups: complete: *p* = 0.03. help to diagnosis: *p* = 0, and teaching tool: *p* = 0). This indicates the heterogeneity of the specific group as different benign lesions can have variably challenging dermoscopic images.

Pigmented lesions, especially lesions with a pigmented network on dermoscopy such as nevi and melanoma, were assessed in the next triad. This triad aimed to test the chatbot to determine if it can recognize a malignant lesion in the comparison between nevi (1st and 3rd descriptions) and melanoma (2nd description). The 2nd description contained the blue-black veil, which is a dermoscopic feature of melanoma [26,27] or pigmented BCC [28]. The differential diagnosis results included all melanocytic lesion subtypes (Table 4).

Taking inflammatory dermatosis images into consideration, a case of psoriasis, sarcoidosis, and alopecia areata was assessed. Psoriasis dermoscopy was presented with the classic dermoscopic finding, with distributed dotted vessels in a reddish pink background and white scales [29]. Additionally, the dermoscopy of sarcoidosis tends to be more challenging in clinical practice, with the orange structureless areas being characteristic of their dermoscopic presentation [30]. Sarcoidosis was not included in the differential diagnosis of the chatbot and was thus evaluated with low marks in completeness (2 ± 0.79) (Table 5). Also, the third description was the first question to the chatbot that included scalp location. The term “an alopecia plaque in the head” was added to that question, specifying the nature of the disease. Statistical significance was found concerning the scores as a dermoscopic image of psoriasis achieved a much higher score in two categories (Table 5) (Kruskal–Wallis test between groups: complete: *p* = 0.02 and teaching tool: *p* = 0.007).

**Figure 2:**
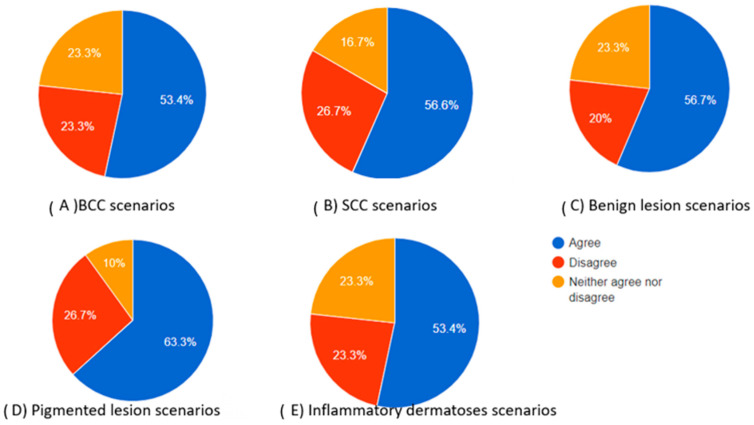
Pie charts showing the percentage of participants who agreed, neither agreed nor disagreed, and disagreed regarding the completeness of the chatbot’s differential diagnosis based on the dermoscopic description for each category of skin lesions.

**Figure 3:**
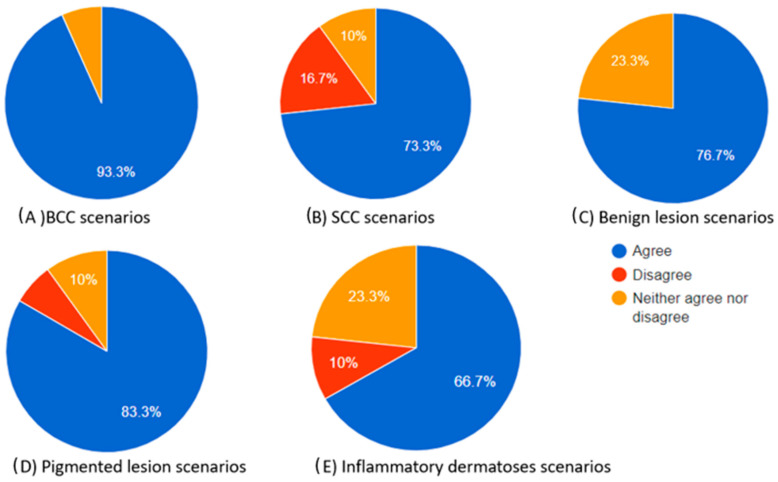
Pie charts illustrating the proportions of participants who agreed, neither agreed nor disagreed, and disagreed regarding the “helpful to diagnosis” category of the chatbot’s differential diagnosis based on the dermoscopic description for each skin lesion.

**Figure 4:**
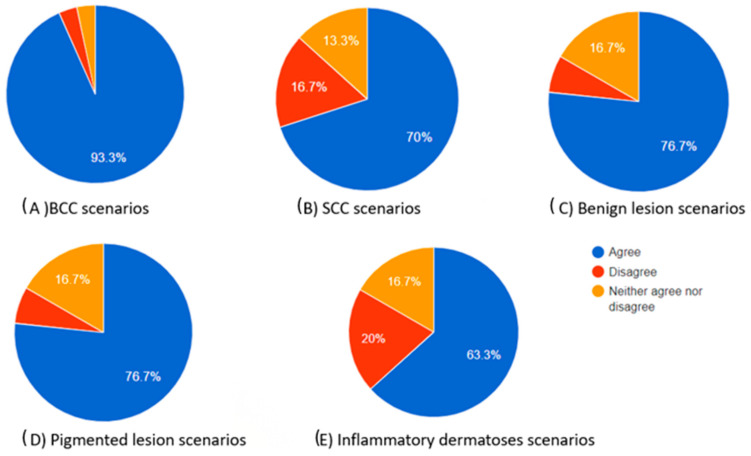
Pie charts showing the proportions of participants who agreed, neither agreed nor disagreed, and disagreed regarding how useful the chatbot’s differential diagnosis teaching tool can be based on the dermoscopic description for each skin lesion.

As indicated by the above evaluations, challenging dermoscopy can occur in the same category of skin lesions. The scores produced cannot be only attributed to the excellence in the differential diagnosis of the chatbot but also to the dermoscopic knowledge of the participant. A clinician who has difficulties in finding the correct diagnosis is more likely to select “neither agree nor disagree”. However, as illustrated in Table 6, which provides a summary of the scores in each category, there was no statistical significance observed in the “complete” category. For the Kruskal–Wallis score in this category between the groups, *p* = 0.881 > 0.05. This suggests that the evaluation of chatbot differential diagnoses remains consistently high, irrespective of the type of skin lesion. As for the diagnosis approach and teaching tool usefulness, BCC scores outperformed SCC scores and inflammatory dermatosis scores (post-hoc Dunn’s tests: *p* = 0.005 and 0 for the “diagnosis” category and *p* = 0.001 and 0 for teaching tool usefulness, respectively). This outcome demonstrates the extensive familiarity dermatologists have with dermoscopic images of BCC while also highlighting the complexity associated with interpreting dermoscopic images of SCC and inflammatory dermatoses. In teaching mode, BCC also outperformed benign and pigmented lesions (*p* = 0.004 both). Inflammatory dermatoses and SCC had the lowest scores in the “teaching tool” category (Table 6). The total percentages of participants who agreed, neither agreed nor disagreed, and disagreed in each skin category are also presented as pie charts in Figure 2, Figure 3 and Figure 4.

Table 6 illustrates the summary of scores in each dermoscopic image triad.

The total percentages of participants who agreed, neither agreed nor disagreed, and disagreed in each skin category are also presented as pie charts in Figure 2, Figure 3 and Figure 4.

Apart from classic descriptions of dermoscopic images, there was a triad of questions that presented signs in dermoscopy with descriptive language and not with their adaptation terms. For example, the so-called rosettes found in actinic keratosis and SCC were presented as four white points arranged as a four-leaf clover. Also, delta signs, commonly found in scabies, were presented as multiple brownish triangular structures. Finally, a strawberry pattern, commonly seen in actinic hyperkeratosis, was described as background erythema interrupted by multiple small keratin-filled follicular ostia [31]. All three categories collected low scores on all the questions (Table 7). This highlights the significance of the information provided by clinicians to the AI program being utilized, such as a chatbot. Moreover, the lower scores may be attributed to the limited dermoscopic information upon which the chatbot seemed to base its diagnostic list. For instance, it appeared that the chatbot determined its diagnoses based on each term provided, such as the colors mentioned. For example, when the term “brownish” was included in the description of the delta sign, it seemed to prompt the chatbot to generate diagnoses associated with dermoscopic features containing this color, often indicative of melanocytic lesions. A similar approach was observed with other responses as well, with terms like “keratin” leading to diagnoses related to seborrheic keratosis. Comparing those findings with the previous summary scores, the descriptive terms did not collect enough points (Kruskal–Wallis testing between all groups with *p* < 0.01).

Table 7 shows three dermoscopic signs with an image-based evaluation. The 1st and 3rd descriptions represent rosettes and strawberry patterns observed in actinic keratosis, while the 2nd dermoscopic image is based on the delta sign seen in scabies.

The subsequent triads integrate dermoscopic images, along with an additional patient characteristic, such as age, phototype, or immune state. This encompasses scenarios comparing patients aged 20 versus 70 years, patients with lighter and darker phototypes, and immunocompetent patients and individuals under immunosuppression (Table 8, Table 9 and Table 10). The differential diagnosis list is adapted to include lesion types appropriate for each category. For instance, in the case of elderly patients, the differential diagnosis included more skin cancer subtypes and lesions like seborrheic and actinic keratosis, which are commonly observed in this demographic [32]. Additionally, atypical spitz nevus, often observed in younger patients and alert clinicians, was also noted [33]. In the third description, the chatbot-generated list remained the same for both the young and old patients (Table 8), indicating that the diagnostic approach adopted by the chatbot remains consistent in some cases. In the case of dark-skinned patients, pigmented lesions such as pigmented BCC, pigmented actinic keratosis, and keratosis papulosa nigra keratosis, as well as skin cancer subtypes commonly reported in black individuals, such as dermatofibrosarcoma and acral melanoma, were more common [34]. In the case of immunosuppression, cases of Kaposi sarcoma, cutaneous lymphoma, and atypical infections were commonly reported, following the incidence of skin diseases in immunosuppressed patients [35].

Table 8 shows the evaluation scores of the chatbot differential diagnosis based on the dermoscopic images plus the characteristics of age.

Table 9 shows the evaluation scores of the chatbot differential diagnosis based on the dermoscopic images plus the characteristics of phenotype.

Table 10 shows the evaluation scores of the chatbot differential diagnosis based on the dermoscopic images plus the characteristics of the immune state.

Completeness of the chatbot differential diagnosis: When dark skin and immune state information were combined with BCC dermoscopy, higher scores were achieved compared to dermoscopic descriptions alone, as well as when combined with age information. However, non-statistically significant results were achieved (Kruskal–Wallis test: *p* = 0.191) between groups with and without such information. For SCC, the dark skin category yielded the highest scores among specific SCC dermoscopic descriptions without statistical significance (*p* = 0.162). Additionally, when focusing on a pigmented lesion description centered around an atypical nevus, no statistically significant differences were observed (*p* = 0.841).

Helpful to diagnosis: BCC descriptions across all categories (with or without additional parameters) received consistently high scores, with no statistically significant differences between them (*p* = 0.1). Incorporating parameters in SCC dermoscopic descriptions resulted in statistically significant higher scores compared to descriptions alone (*p* = 0.040), especially between simple descriptions and descriptions with skin type data (*p* = 0.004). A similar trend was not observed for pigmented lesions (*p* = 0.052).

Teaching tool usefulness: All categories and lesions received high scores, with few exceptions such as SCC and pigmented lesions in dark-skinned patients and SCC in patients with immunosuppression. No statistically significant difference was reported between the groups (BCC: *p* = 0.052, SCC: *p* = 0.097, and pigmented lesion: *p* = 0.997).

## 4. Discussion

This survey marks the initial attempt to evaluate the comprehensibility of the dermoscopic language by a chatbot, shedding light on the interaction between dermatologists and AI programs in this specific realm of dermatology. Even among dermatologists, the language used in dermoscopy can be perplexing as descriptive and metaphorical languages are occasionally employed, leading to a subjective interpretation of the observed image [31]. Furthermore, the diagnostic utility of a dermoscopic image may vary based on the equipment accessible and the expertise of each participant. Hence, dermoscopy presents a challenging subject for assessment when integrating AI technologies.

The first step of our study was to evaluate the differential diagnosis list when examples of different skin lesions were presented. The examples included dermoscopic descriptions of skin cancers such as BCC, SCC, and melanoma, skin cancer mimickers such as actinic and seborrheic keratosis, dermatofibroma, and atypical nevus, and inflammatory dermatoses such as psoriasis and alopecia areata. Each participant evaluated each description on a scale of 1 to 3 based on how complete, helpful to diagnosis, and educational the given differential list was.

It is worth mentioning that when the chatbot was asked about a differential diagnosis, except for the list presented in the previous tables, it reported an accompanying text. This text usually presented extra features to look for when assessing a lesion that could lead to the diagnosis. For example, in the case of a psoriasis description, the original question to the chatbot was “what is the differential diagnosis of a lesion whose dermoscopy includes distributed dotted vessels in a reddish pink background and white scales”. The chatbot began to generate its diagnosis list, assuming a probability priority. Additionally, it associated other diagnoses with additional characteristics that, when combined with the provided description, may alter the diagnosis while prompting the user to seek out those characteristics. For instance, it links a violaceous background with lichen planus [36], a central white scar-like area with dermatofibroma [37], and ulceration with Bowen’s disease [38]. As a result, it creates an algorithm based on the description given and potentially newly discovered characteristics.

Questions regarding slight changes in the questions to the chatbot could lead to a different differential diagnosis. The dermoscopic language has its own vocabulary; therefore, synonyms can be used. For example, in the case of BCC, instead of “arborizing”, the term “branching” can be used to describe the vessels of the skin cancer. In this case, a similar differential diagnosis is produced (BCC, SCC, melanoma, etc.). The same occurs if a non-dermoscopic term is replaced and another adjective is used. For example, if the term “scattered” is used to describe the dotted vessels in the psoriasis dermoscopy, the chatbot maintains a similar set of potential diagnoses (psoriasis, lichen planus, and pityriasis rosea).

Furthermore, all the responses to the user concluded with a message advocating for a visit to a clinician or a specialized dermatologist, along with highlighting the likelihood of undergoing a biopsy for the ultimate diagnosis.

Completeness: In each case, the chatbot produced a long list of differential diagnoses, scoring low in the case of SCC and inflammatory dermatoses but not with statistical significance, meaning that the participants were almost equally satisfied with the answers given. The scores were reduced in the case of practical descriptions of dermoscopic signs, such as the description of the delta sign as multiple brownish triangular structures and the strawberry pattern as background erythema interrupted by multiple small keratin-filled follicular ostia. In those cases, the chatbot did not produce a satisfactory answer, maybe due to the subjective use of the dermoscopic language and the lack of key dermoscopy words that could easily reveal the answer. Incorporating patient information such as age, phototype, and immune state did not alter the scores in the BCC and pigmented lesion categories, but it did slightly increase scores for SCC without reaching statistical significance. This suggests that in complex cases, clinicians may require additional available information.

Diagnosis approach: The diagnostic approach category garnered more points compared to the complete category. Additionally, we posit that presenting the entire response to participants, including the added characteristics to consider for assessing alternative diagnoses, would likely result in higher scores. In this instance, the scores for BCC surpassed those for SCC and inflammatory dermatoses (post-hoc Dunn’s tests: *p* = 0.005 and 0 for the diagnosis category). This outcome underscores dermatologists’ extensive familiarity with dermoscopic images of BCC while also underscoring the challenges associated with interpreting dermoscopic images of SCC and inflammatory dermatoses. Incorporating patient information such as age, phototype, and immune state did not alter the scores in most cases. However, the diagnostic list generated from the description of SCC in a dark-skinned patient received more points than the description of SCC alone. This may be because SCC in dark-skinned patients is rare and may exhibit unique dermoscopic characteristics [39]. Additionally, the list generated in this case adhered to the epidemiology of cancerous lesions in dark-skinned patients [40], potentially further satisfying the participants.

Teaching method: In this category, the chatbot answers collected the most points, indicating that AI can not only help in clinical practice but can also be a valuable educational tool. As in the diagnosis category, the scores for BCC surpassed those for SCC and inflammatory dermatoses (post-hoc Dunn’s tests: *p* = 0.001 and 0 for teaching tool usefulness, respectively). It is worth noting that the scores for the “teaching” method usually aligned with the scores for the “helpful to diagnosis” method, indicating that participants believe that a tool that aids in diagnosis can be highly educational for medical students and new clinicians. In cases where patients’ demographic data were added, SCC and pigmented lesions in dark-skinned patients and SCC in patients with immunosuppression received the lowest scores, but no statistical significance between groups was reported. This proves that adding characteristics of the patients did not alter the quality of the chatbot’s answers.

A study with a similar method evaluated the ability of chatbots to recognize the dermatopathological language, similar to how we assessed the dermoscopic language [13]. Dermatopathological language as a dermoscopic language uses its own specialized vocabulary and terminology, including cell types, cancer infiltration levels, and immunohistochemical stains. In our study, the chatbots were assessed by experts regarding completeness, accuracy, ease of understanding, the possibility of causing stress to patients, and the likelihood of causing harm. This study is more focused on the patient’s use of chatbots. It is worth mentioning that BCC received one of the highest scores for the “ease of understanding” category, and melanoma scored lowest in the “likelihood to cause harm” category. It is important to recognize that dermoscopy and dermatopathology use different terminologies, resulting in varying levels of chatbot comprehension regarding the same diagnosis.

Chatbots and, generally, AI apps, as indicated by the abovementioned results, can offer support, particularly in scenarios where a clinician might overlook a diagnosis in a dermoscopic image requiring assessment. This collaboration becomes crucial in situations where dermoscopic expertise is limited, time is constrained, or when handling a large volume of cases. Thus, apart from focusing on comparing clinicians and AI applications, emphasis should be placed on the outcomes of their collaboration. Chatbots serve as beneficial tools in healthcare settings, and they cannot substitute for the expertise and judgment of trained medical specialists [8].

Limitations: Our study has several limitations that warrant acknowledgment. First, it relies on the ChatGPT3.5 chatbot, chosen due to its accessibility to the general population. However, employing more advanced chatbots could yield different results. Second, the differential diagnosis list in each case is derived from the initial response generated by the chatbot. Also, the answers depend on the input information related to dermoscopic knowledge. Considering that subsequent responses may vary slightly, this could potentially impact the outcomes. Finally, the survey was conducted among dermatologists for evaluation, indicating potential variations in participants’ levels of experience and dermoscopic knowledge.

## 5. Conclusions

The AI chatbot underwent testing to provide diagnoses based on the dermoscopic language, and it performed well, generating mostly comprehensive, diagnostically helpful, and educational lists of differential diagnoses. The evaluation of the chatbot was conducted using dermoscopic descriptions of skin cancers like BCC, SCC, and melanoma, skin cancer mimickers such as actinic and seborrheic keratosis, dermatofibroma, and atypical nevus, and inflammatory dermatoses like psoriasis and alopecia areata. Lower scores were observed for the AI chatbot in more challenging cases of SCC and inflammatory dermatoses and in the assessment of subjective descriptions of dermoscopic signs. Furthermore, by incorporating patient characteristics such as age, phototype, or immune state, the differential diagnosis list in each case was tailored to include lesion types suitable for each category, highlighting the AI’s adaptability in assessing diagnoses and making it a valuable aid for dermatologists.

## Figures and Tables

**Table 1:** BCC dermoscopy-based image scores according to the evaluation of dermatologists who completed the questionnaire. The exact scores of the triad of the BCC dermoscopy-based images are as follows: **1st description:** Complete: 60% Agree, 10% Neither agree nor disagree, and 30% Disagree, Total score: 2.3 ± 0.91, Helpful to diagnosis: 100% Agree, Total score: 3, Teaching tool: 100% agree, and Total score: 3; **2nd description**: Complete: 60% Agree, 30% Neither agree nor disagree, and 10% Disagree, Total score: 2.5 ± 0.68, Helpful to diagnosis: 90% Agree and 10% Neither agree nor disagree, Total score: 2.9 ± 0.31, Teaching tool: 90% Agree and 10% Neither agree nor disagree, and Total score: 2.9 ± 0.31; and **3rd description**: Complete: 40% Agree, 30% Neither agree nor disagree, and 30% Disagree, Total score: 2.1 ± 0.85, Helpful to diagnosis: 90% Agree and 10% Disagree, Total score: 2.8 ± 0.61, Teaching tool: 90% Agree and 10% Disagree, and Total score: 2.8 ± 0.61.

Original Question to the Chatbot: What Is the Differential Diagnosis of a Lesion Whose Dermoscopic Image Includes the Following?	Differential Diagnosis Generated	Complete	Helpful to Diagnosis	Teaching Tool
1 (A) Arborizing vessels, (B) ulceration, (C) shiny white-red structureless areas, and (D) short white streaks	Basal cell carcinoma (BCC)	2.3 ± 0.91	3	3
	Squamous cell carcinoma (SCC)			
	Amelanotic melanoma			
	Pyogenic granuloma			
	Other dermatological conditions			
2 (A) Ulceration, (B) multiple small erosions, (C) shiny white-red structureless areas, and (D) shiny white lines.	Squamous cell carcinoma (SCC)	2.5 ± 0.68	2.9 ± 0.31	2.9 ± 0.31
	Basal cell carcinoma (BCC)			
	Pyogenic granuloma			
	Infectious or inflammatory dermatoses			
	Traumatic lesions			
3 (A) Arborizing vessels, (B) blue-grey ovoid nests (large clods), (C) ulceration, and (D) peppering (grey or brown dots) and /or clods.	Melanoma	2.1 ± 0.85	2.8 ± 0.61	2.8 ± 0.61
	Atypical nevus (dysplastic nevus)			
	Basal cell carcinoma (BCC)			
	Seborrheic keratosis			
	Dermatofibroma			
	Eccrine poroma			
BCC dermoscopy-based images (Sum)		2.3 ± 0.83	2.9 ± 0.4	2.9 ± 0.4

**Table 2:** SCC dermoscopy-based image scores according to the evaluation of dermatologists who completed the questionnaire. The exact scores of the triad of the SCC dermoscopy-based images are as follows: **1st description**: Complete: 50% Agree, 20% Neither agree nor disagree, and 30% Disagree, Total score: 2.1 ± 0.85, Helpful to diagnosis: 60% Agree, 20% Neither agree nor disagree, and 20% Disagree, Total score: 2.4 ± 0.81, Teaching tool: 50% Agree, 30% Neither agree nor disagree, and 20% Disagree, and Total score: 2.3 ± 0.79; **2nd description:** Complete: 50% Agree, 20% Neither agree nor disagree, and 30% Disagree, Total score: 2.2 ± 0.89, Helpful to diagnosis: 80% Agree, 10% Neither agree nor disagree, and 10% Disagree, Total score: 2.7 ± 0.65. Teaching tool: 80% Agree, 10% Neither agree nor disagree, and 10% Disagree, and Total score: 2.7 ± 0.65; and **3rd description**: Complete: 70% Agree, 10% Neither agree nor disagree, and 20% Disagree, Total score: 2.5 ± 0.82, Helpful to diagnosis: 80% Agree and 20% Disagree, Total Score: 2.9 ± 0.3, Teaching tool: 80% Agree and 20% Disagree, and Total score: 2.9 ± 0.3.

Original Question to the Chatbot: What Is the Differential Diagnosis of a Lesion Whose Dermoscopic Image Includes the Following?	Differential Diagnosis Generated	Complete	Helpful to Diagnosis	Teaching Tool
1 (A) Brown or grey dots, (B) pink or skin-colored eccentric structureless areas, and (C) glomerular (coiled) vessels	Lentigo	2.1 ± 0.85	2.4 ± 0.81	2.3 ± 0.79
	Seborrheic keratosis			
	Melanocytic nevus			
	Dermatofibroma			
	Amelanotic melanoma			
	Angiokeratoma			
2 (A) A scaly surface, (B) small brown globules, (C) linear greyish dots, and (D) homogeneous pigmentation	Squamous cell carcinoma (SCC)	2.2 ± 0.89	2.7 ± 0.65	2.7 ± 0.65
	Bowen’s disease (squamous cell carcinoma in situ)			
	Lentigo maligna			
	Actinic keratosis			
	Pigmented basal cell carcinoma (BCC)			
	Sebaceous hyperplasia			
3 (A) White circles, (B) keratin, (C) pink background, and (D) blood spots	Seborrheic keratosis	2.5 ± 0.82	2.9 ± 0.3	2.9 ± 0.3
	Squamous cell carcinoma (SCC)			
	Bowen’s disease			
	Verruca vulgaris (common wart)			
	Pyogenic granuloma			
	Amelanotic melanoma			
SCC dermoscopy-based images (Sum)		2.3 ± 0.86	2.6 ± 0.66	2.6 ± 0.67

**Table 3:** Benign lesion dermoscopy-based image scores according to the evaluation of dermatologists who completed the questionnaire. The exact scores of the triad are as follows: **1st description (actinic keratosis):** Complete: 40% Agree, 30% Neither agree nor disagree, and 30% Disagree, Total score: 2.1 ± 0.84, Helpful to diagnosis: 50% Agree and 50% Neither agree nor disagree, Total score: 2.5 ± 0.5, Teaching tool: 50% Agree, 40% Neither agree nor disagree, and 10% Disagree, and Total score: 2.4 ± 0.67; **2nd description (seborrheic keratosis)**: Complete: 60% Agree, 20% Neither agree nor disagree, and 20% Disagree, Total score: 2.4 ± 0.81, Helpful to diagnosis: 80% Agree and 20% Neither agree nor disagree, Total score: 2.8 ± 0.4. Teaching tool: 80% Agree, 10% Neither agree nor disagree, and 10% Disagree, and Total score: 2.7 ± 0.65; and **3rd description (dermatofibroma):** Complete: 70% Agree, 20% Neither agree nor disagree, and 10% Disagree, Total score: 2.6 ± 0.67, Helpful to diagnosis: 100% Agree, Total score: 3, Teaching tool: 100% Agree, and Total score: 3.

Original Question to the Chatbot: What Is the Differential Diagnosis of a Lesion Whose Dermoscopic Image Includes the Following?	Differential Diagnosis Generated	Complete	Helpful to Diagnosis	Teaching Tool
1 (A) A pigmented pseudo-network and (B) widened follicular openings along with (C) fine scaling	Seborrheic keratosis	2.1 ± 0.84	2.5 ± 0.5	2.4 ± 0.67
	Lichen planus			
	Psoriasis			
	Lichen simplex chronicus			
	Pityriasis rosea			
	Basal cell carcinoma (BCC)			
	Pigmented actinic keratosis			
2 Fissures and ridges giving a cerebriform appearance	Seborrheic keratosis	2.4 ± 0.81	2.8 ± 0.4	2.7 ± 0.65
	Dermatosis papulosa nigra (DPN)			
	Verruca vulgaris (common wart)			
	Squamous cell carcinoma (SCC)			
	Keratoacanthoma			
	Giant congenital melanocytic nevus			
3 A peripheral delicate pigment network and central white patch	Melanocytic nevus	2.6 ± 0.67	3	3
	Dermatofibroma			
	Pigmented basal cell carcinoma (BCC)			
	Seborrheic keratosis			
	Lentigo maligna			
	Post-inflammatory hypopigmentation			
	Halo nevus			
	Other melanocytic lesions			
Benign lesion dermoscopy-based images (Sum)		2.4 ± 0.8	2.8 ± 0.43	2.7 ± 0.59

**Table 4:** Pigmented lesion dermoscopy-based image scores according to the evaluation of dermatologists who completed the questionnaire. The exact scores of the triad of the pigmented lesion dermoscopy-based images are as follows: **1st description:** Complete: 70% Agree and 30% Disagree, Total score: 2.2 ± 0.89, Helpful to diagnosis: 90% Agree and 10% Disagree, Total score: 2.7 ± 0.65, Teaching tool: 80% Agree, 10% Neither agree nor disagree, and 10% Disagree, and Total score: 2.6 ± 0.67; **2nd description:** Complete: 70% Agree, 10% Neither agree nor disagree, and 20% Disagree, Total score: 2.5 ± 0.82, Helpful to diagnosis: 80% Agree and 20% Neither agree nor disagree, Total score: 2.8 ± 0.4. Teaching tool: 80% Agree and 20% Neither agree nor disagree, and Total score: 2.8 ± 0.4; and **3rd description:** Complete: 50% Agree, 20% Neither agree nor disagree, and 30% Disagree, Total score: 2.2 ± 0.91, Helpful to diagnosis: 80% Agree, 10% Neither agree nor disagree, and 10% Disagree, Total score: 2.7 ± 0.65, Teaching tool: 70% Agree, 20% Neither agree nor disagree, and 10% Disagree, and Total score: 2.8 ± 0.4.

Original Question to the Chatbot: What Is the Differential Diagnosis of a Lesion Whose Dermoscopic Image Includes the Following?	Differential Diagnosis Generated	Complete	Helpful to Diagnosis	Teaching Tool
1 (A) An atypical pigment network, asymmetry in structure or color, (B) brown dots, and (C) irregular dots and globules	Melanoma	2.2 ± 0.89	2.7 ± 0.65	2.6 ± 0.67
	Atypical nevus			
	Basal cell carcinoma (BCC)			
	Squamous cell carcinoma (SCC)			
	Seborrheic keratosis			
	Pigmented actinic keratosis			
	Atypical fibroxanthoma			
	Other malignancies			
2 (A) A blue-white veil, (B) multiple brown dots, and (C) pseudopods	Melanoma	2.5 ± 0.82	2.8 ± 0.4	2.8 ± 0.4
	Basal cell carcinoma (BCC)			
	Pigmented actinic keratosis			
	Seborrheic keratosis			
	Dermatofibroma			
	Spitzoid lesions			
3 (A) Structureless hyperpigmented areas in the center and (B) reticular at the periphery	Melanocytic nevus	2.2 ± 0.91	2.7 ± 0.65	2.8 ± 0.4
	Lentigo			
	Dysplastic nevus			
	Melanoma			
	Pigmented basal cell carcinoma (BCC)			
	Pigmented Bowen’s disease (squamous cell carcinoma in situ)			
	Amelanotic melanoma			
Pigment network dermoscopy lesion-based images		2.4 ± 0.88	2.8 ± 0.48	2.7 ± 0.59

**Table 5:** Inflammatory dermatosis dermoscopy-based image scores according to the evaluation of dermatologists who completed the questionnaire. The exact scores of the inflammatory dermatosis dermoscopy-based images are as follows: **1st description (psoriasis**): Complete: 80% Agree, 10% Neither agree nor disagree, and 10% Disagree, Total score: 2.7 ± 0.65, Helpful to diagnosis: 80% Agree, 10% Neither agree nor disagree, and 10% Disagree, Total score: 2.7 ± 0.65, Teaching tool: 80% Agree, 10% Neither agree nor disagree, and 10% Disagree, and Total score: 2.7± 0.65; **2nd description (sarcoidosis):** Complete: 30% Agree, 40% Neither agree nor disagree, and 30% Disagree, Total score: 2 ± 0.79, Helpful to diagnosis: 50% Agree, 40% Neither agree nor disagree, and 10% Disagree, Total score: 2.4 ± 0.67, Teaching tool: 40% Agree, 30% Neither agree nor disagree, and 30% Disagree, Total score: 2.1 ± 0.85; and **3rd description (alopecia areata):** Complete: 50% Agree, 20% Neither agree nor disagree, and 30% Disagree, Total score: 2.2 ± 0.89, Helpful to diagnosis: 70% Agree, 20% Neither agree nor disagree, and 10% Disagree, Total score: 2.6 ± 0.67, Teaching tool: 70% Agree, 10% Neither agree nor disagree, and 20% Disagree, and Total score: 2.5 ± 0.82.

Original Question to the Chatbot: What Is the Differential Diagnosis of a Lesion Whose Dermoscopic Image Includes the Following?	Differential Diagnosis Generated	Complete	Helpful to Diagnosis	Teaching Tool
1 Distributed dotted vessels in a reddish pink background and white scales	Psoriasis	2.7 ± 0.65	2.7 ± 0.65	2.7 ± 0.65
	Lichen planus			
	Discoid lupus erythematosus (DLE)			
	Pityriasis rosea			
	Tinea corporis (ringworm)			
	Basal cell carcinoma (BCC)			
	Bowen’s disease			
2 Orange structureless areas with overlying focused linear vessels and white areas	Seborrheic keratosis (SK)	2 ± 0.79	2.4 ± 0.67	2.1 ± 0.85
	Lichen planus			
	Basal cell carcinoma (BCC)			
	Angioma			
	Hemangioma			
	Actinic keratosis (AK)			
	Bowen’s disease (squamous cell carcinoma in situ)			
	Keratoacanthoma			
	Amelanotic melanoma			
	Dermatofibroma			
3 Yellow and black dots and at least one broken hair	Alopecia areata	2.2 ± 0.89	2.6 ± 0.67	2.5 ± 0.82
	Tinea capitis			
	Trichotillomania			
	Discoid lupus erythematosus (DLE)			
	Central centrifugal cicatricial alopecia (CCCA)			
	Secondary cicatricial alopecia			
	Folliculitis decalvans			
Inflammatory dermatosis dermoscopy-based images		2.3 ± 0.82	2.6 ± 0.67	2.4 ± 0.81

**Table 6:** The table summarizes the scores for each dermoscopic image triad.

	Complete	Helpful to Diagnosis	Teaching Tool Usefulness
BCC dermoscopy-based images (Sum)	2.3 ± 0.83	2.9 ± 0.4	2.9 ± 0.4
SCC dermoscopy-based images (Sum)	2.3 ± 0.86	2.6 ± 0.66	2.6 ± 0.67
Benign lesion dermoscopy-based images (Sum)	2.4 ± 0.8	2.8 ± 0.43	2.7 ± 0.59
Pigment network dermoscopy lesion-based images (Sum)	2.4 ± 0.88	2.8 ± 0.48	2.7 ± 0.59
Inflammatory dermatosis dermoscopy-based images (Sum)	2.3 ± 0.82	2.6 ± 0.67	2.4 ± 0.81

**Table 7:** Table presenting cases based on dermoscopic signs.

Original Question to the Chatbot: What Is the Differential Diagnosis of a Lesion Whose Dermoscopic Image Includes the Following?	Differential Diagnosis Generated	Complete	Helpful to Diagnosis	Teaching Tool
Four white points arranged as a four-leaf clover	Dermatofibroma	1.7 ± 0.79	2 ± 0.79	2 ± 0.79
	Seborrheic keratosis			
	Lichen planus-like keratosis (LPLK)			
	Angiokeratoma			
	Basal cell carcinoma (BCC)			
	Melanocytic lesions			
Multiple brownish triangular structures creating a delta sign	Melanocytic nevus	1.6 ± 0.76	1.9 ± 0.84	2 ± 0.79
	Reed nevus			
	Spitzoid lesions			
	Lentigo			
	Melanoma			
Background erythema interrupted by multiple small keratin-filled follicular ostia	Seborrheic keratosis	1.7 ± 0.92	2.2 ± 0.76	2.1 ± 0.84
	Dermatosis papulosa nigra (DPN)			
	Angioma serpiginosum			
	Lichen planus-like keratosis (LPLK)			
	Follicular keratosis			
	Basal cell carcinoma (BCC)			
Signs and descriptions (Sum)		1.7 ± 0.82	2 ± 0.8	2 ± 0.8

**Table 8:** Table displaying the evaluation scores for the chatbot’s differential diagnosis, considering dermoscopic images and age characteristics.

Dermoscopy Category	Original Question to the Chatbot: What Is the Differential Diagnosis of a Lesion Whose Dermoscopic Image Includes the Following?	Differential Diagnosis Generated (20 Years)	Differential Diagnosis Generated (70 Years Old)	Complete	Helpful to Diagnosis	Teaching Tool
BCC	(A) Arborizing vessels, (B) ulceration, (C) shiny white-red structureless areas, and (D) short white streaks	Melanoma	Melanoma	2.3 ± 0.79	2.8 ± 0.61	2.7 ± 0.65
		Atypical spitz nevus	Basal cell carcinoma (BCC)			
		Basal cell carcinoma (BCC)	Squamous cell carcinoma a (SCC)			
		Seborrheic keratosis	Amelanotic melanoma			
		Amelanotic melanoma	Pyogenic granuloma			
		Pyogenic granuloma	Seborrheic keratosis			
		Nodular melanoma	Actinic keratosis			
SCC	(A) Brown or grey dots, (B) pink or skin-colored eccentric structureless areas, and (C) glomerular (coiled) vessels	Melanocytic nevus	Seborrheic keratosis	2.2 ± 0.76	2.6 ± 0.67	2.6 ± 0.67
		Seborrheic keratosis	Lentigo			
		Lentigo	Melanocytic nevus			
		Dermatofibroma	Dermatofibroma			
		Angiokeratoma	Angiokeratoma			
		Amelanotic melanoma	Amelanotic melanoma			
Pigmented lesion	An atypical pigment network, asymmetry in structure or color, brown dots, and irregular dots and globules	Melanoma	Melanoma	2.3 ± 0.92	2.7 ± 0.65	2.7 ± 0.65
		Atypical nevus	Atypical nevus			
		Basal cell carcinoma (BCC)	Basal cell carcinoma (BCC)			
		Squamous cell carcinoma (SCC)	Squamous cell carcinoma (SCC)			
		Seborrheic keratosis	Seborrheic keratosis			
		Pigmented actinic keratosis	Pigmented actinic keratosis			
		Lentigo maligna	Lentigo maligna			

**Table 9:** Table displaying the evaluation scores for the chatbot’s differential diagnosis, considering dermoscopic images and phenotypic characteristics.

Dermoscopy Category	Original Question to the Chatbot: What Is the Differential Diagnosis of a Lesion Whose Dermoscopic Image Includes the Following?	Differential Diagnosis Generated (without Any Characteristics)	Differential Diagnosis Generated (with Darker Phenotypes)	Complete	Helpful to Diagnosis	Teaching Tool
BCC	(A) Arborizing vessels, (B) ulceration, (C) shiny white-red structureless areas, and (D) short white streaks	Basal cell carcinoma (BCC) Squamous cell carcinoma (SCC) amelanotic melanoma Pyogenic granuloma Other dermatological conditions	Melanoma Basal cell carcinoma (BCC) Squamous cell carcinoma (SCC) Hemangioma Pigmented basal cell carcinoma (PBCC) Dermatofibroma Traumatic or inflammatory lesions	2.6 ± 0.67	2.9 ± 0.31	2.9 ± 0.31
SCC	(A) Brown or grey dots, (B) pink or skin-colored eccentric structureless areas, and (C) glomerular (coiled) vessels	Lentigo Seborrheic keratosis Melanocytic nevus dermatofibroma amelanotic melanoma Angiokeratoma	Seborrheic keratosis Dermatosis papulosa nigra Angiokeratoma LichenpPlanus Acral lentiginous Melanoma Pigmented basal cell carcinoma (PBCC)	2.5 ± 0.82	2.9 ± 0.31	2.7 ± 0.65
Pigmented lesion	An atypical pigment network, asymmetry in structure or color, brown dots, and irregular dots and globules	Melanoma Atypical nevus Basal cell carcinoma (BCC) Squamous cell carcinoma (SCC) Seborrheic keratosis Pigmented actinic keratosis Atypical fibroxanthoma Other malignancies	Melanoma Atypical nevus Basal cell carcinoma (BCC) Squamous cell carcinoma (SCC) Seborrheic keratosis Pigmented actinic keratosis Lentigo maligna	2.5 ± 0.82	2.7 ± 0.65	2.6 ± 0.67

**Table 10:** Table displaying the evaluation scores for the chatbot’s differential diagnosis, taking into account dermoscopic images and immune status characteristics.

Dermoscopy Category	Original Question to the Chatbot: What Is the Differential Diagnosis of a Lesion Whose Dermoscopic Image Includes the Following?	Differential Diagnosis Generated (without Any Characteristics)	Differential Diagnosis Generated (with Immunosuppression)	Complete	Helpful to Diagnosis	Teaching Tool
BCC	(A) Arborizing vessels, (B) ulceration, (C) shiny white-red structureless areas, and (D) short white streaks	Basal cell carcinoma (BCC) Squamous cell carcinoma (SCC) Amelanotic melanoma Pyogenic granuloma Other dermatological conditions	Cutaneous lymphoma Kapos sarcoma Atypical infections Aggressive squamous cell carcinoma (SCC) Atypical melanoma Merkel cell carcinoma Reactivation of viral infections	2.7 ± 0.65	3	2.7 ± 0.65
SCC	(A) Brown or grey dots, (B) pink or skin-colored eccentric structureless areas, and (C) glomerular (coiled) vessels	Lentigo Seborrheic keratosis Melanocytic nevus Dermatofibroma amelanotic melanoma Angiokeratoma	Kaposi sarcoma Cutaneous lymphoma Atypical infections Aggressive squamous cell carcinoma (SCC) Atypical melanoma Merkel cell carcinoma	2.2 ± 0.76	2.7 ± 0.47	2.5 ± 0.51
Pigmented lesion	An atypical pigment network, asymmetry in structure or color, brown dots, and irregular dots and globules	Melanoma Atypical nevus Basal cell carcinoma (BCC) Squamous cell carcinoma (SCC) Seborrheic keratosis Pigmented actinic keratosis Atypical fibroxanthoma Other malignancies	Melanoma Atypical nevus Basal cell carcinoma (BCC) Squamous cell carcinoma (SCC) Kaposi sarcoma Cutaneous lymphoma Atypical infections	2.5 ± 0.68	3	2.8 ± 0.4

## Data Availability

The data described in this study are available upon request from the corresponding author.
